# Host cell type-dependent translocation and PhoP-mediated positive regulation of the effector SseK1 of *Salmonella enterica*

**DOI:** 10.3389/fmicb.2015.00396

**Published:** 2015-04-29

**Authors:** Fernando Baisón-Olmo, María Galindo-Moreno, Francisco Ramos-Morales

**Affiliations:** Departamento de Genética, Facultad de Biología, Universidad de SevillaSevilla, Spain

**Keywords:** *Salmonella*, SseK1, type III secretion, PhoQ/PhoP two-component system, epithelial cells, macrophages, fibroblasts, bioluminescence

## Abstract

*Salmonella enterica* expresses two virulence-related type III secretion systems (T3SSs) encoded in *Salmonella* pathogenicity island 1 (SPI1) and SPI2, respectively. SseK1 is a poorly characterized substrate of the SPI2-encoded T3SS. Here, we show that this effector is essential to get full virulence both in oral and intraperitoneal mice infections, in spite of not having a role in invasion or intracellular proliferation in cultured mammalian cells. *In vitro*, expression of *sseK1* was higher in media mimicking intracellular conditions, when SPI2 was induced, but it was also significant under SPI1 inducing conditions. A detailed analysis of translocation of SseK1 into host cells unveiled that it was a substrate of both, T3SS1 and T3SS2, although with different patterns and kinetics depending on the specific host cell type (epithelial, macrophages, or fibroblasts). The regulation of the expression of *sseK1* was examined using *lacZ* and bioluminescent *lux* fusions. The two-component system PhoQ/PhoP is a positive regulator of this gene. A combination of sequence analysis, directed mutagenesis and electrophoretic mobility shift assays showed that phosphorylated PhoP binds directly to the promoter region of *sseK1* and revealed a PhoP binding site located upstream of the predicted -35 hexamer of this promoter.

## Introduction

*Salmonella enterica* is a leading cause of bacterial foodborne infections worldwide that can induce from enterocolitis to systemic diseases, depending on the serovar-host combination (Chen and Jiang, 2014). The broad-host-range serovar Typhimurium causes gastroenteritis in humans, calves and other animals, but it causes a systemic typhoid fever-like disease in susceptible mouse strains (Tsolis et al., 1999; Uzzau et al., 2000). The virulence of these bacteria relies on the possession of specific genes. Many of them are horizontally transferred elements that are clustered in *Salmonella* pathogenicity islands (SPIs; Gyles and Boerlin, 2014). The biggest and best studied clusters are SPI1 and SPI2, which encode two type III secretion systems, T3SS1 and T3SS2, that are important for invasion of non-phagocytic cells and for intracellular survival and proliferation, respectively (Galán and Curtiss, 1989; Ochman et al., 1996; Shea et al., 1996). These are flagellum-like one-step transport systems that carry out translocation of proteins, known as effectors, across the two membranes of Gram-negative bacteria and the host cell membrane.

More than 30 effectors are secreted through *Salmonella* T3SSs (Ramos-Morales, 2012; Habyarimana et al., 2014). Some of them are encoded in SPI1 or SPI2 but many are encoded outside the islands. The best characterized *Salmonella* invasion mechanism, the “trigger” mechanism, requires at least six T3SS1 effectors, SipA, SipC, SopB, SopE, SopE2, and SptP, that induce remodeling of actin cytoskeleton (Guiney and Lesnick, 2005). SipA, SopB, SopE, and SopE2 are also involved in the disruption of epithelial tight junctions (Boyle et al., 2006), whereas AvrA is a tight junction stabilizer (Liao et al., 2008). SopA, another T3SS1 effector, is involved in *Salmonella*-induced polymorphonuclear leukocytes transepithelial migration (Zhang et al., 2006). Other processes where T3SS1 is involved are the early and intermediate stages of the *Salmonella*-containing vacuole (SCV) biogenesis (Bakowski et al., 2008; Steele-Mortimer, 2008), and the induction of a rapid form of pyroptosis, a caspase-1 dependent form of programmed cell death, in macrophages (Fink and Cookson, 2007). T3SS2 is expressed intracellularly in response to the low pH and nutrient concentration found in the lumen of the SCV. This system translocates more than 20 effectors through the SCV membrane and is involved in several processes including intermediate and late stages of the SCV biogenesis, generation of tubular networks (Schroeder et al., 2011), apoptosis in epithelial cells, and delayed pyroptosis in macrophages (Fink and Cookson, 2007).

According to the different predominant functions related to T3SS1 and T3SS2, the conditions for optimal expression of SPI1 or SPI2 are reached at different moments of the infection. These conditions can be modeled *in vitro* using appropriate media: rich medium with low aeration and high NaCl concentration for SPI1, and minimal medium with low pH and low Mg^2+^ concentration for SPI2. Some effectors are specifically coexpressed with their cognate T3SS. There are, however, other effectors that are expressed under a broad range of conditions and can be secreted through both systems. This has been described for GtgE (Niemann et al., 2011), PipB2 (Baisón-Olmo et al., 2012), SlrP (Miao and Miller, 2000; Cordero-Alba and Ramos-Morales, 2014), SopD (Jones et al., 1998; Brumell et al., 2003), SpvC (Mazurkiewicz et al., 2008; Haneda et al., 2012), SpvD (Niemann et al., 2011), SspH1 (Miao et al., 1999), SteA (Cardenal-Muñoz and Ramos-Morales, 2011), and SteE (Niemann et al., 2011).

SseK1 was identified in *S. enterica* serovar Typhimurium as a T3SS substrate because of its similarity to known secreted proteins from enterohemorrhagic *Escherichia coli* and *Citrobacter rodentium* (Kujat Choy et al., 2004). Translocation of this protein into epithelial cells was shown to be T3SS2-dependent and after translocation SseK1 localized to the host cytosol. At least two paralogs exist in some *S. enterica* serovars or strains: SseK2 (Kujat Choy et al., 2004), which shares 61% amino acid sequence identity with SseK1, and SseK3 (Brown et al., 2011), which is encoded in a prophage and is 75% identical to SseK2. Because of their striking similarity they are considered members of the same effector family and they are predicted to have redundant functions. However, the specific roles of these proteins in the host cells are unknown and there are conflicting reports about their relevance for intracellular replication of *Salmonella* and for virulence in mice. A study of the contribution of some T3SS2 effectors to replication in host cells reported that a triple mutant *sseK1 sseK2 sseK3* had significantly reduced growth levels in RAW264.7 macrophages but showed no defect in bacterial counts in systemic organs of mice after oral infection (Buckner et al., 2011). In contrast, another study showed significant attenuation for this mutant in mice but did not detect intracellular growth defects (Brown et al., 2011).

In this work, we carry out a detailed analysis of the patterns of expression and the kinetics of translocation of SseK1 into different host cell models. Our data suggest that, under physiological conditions of expression, SseK1 is not translocated upon the initial contact with the eukaryotic cell but when *Salmonella* is inside the cell. Interestingly, translocation can occur through T3SS1 and/or T3SS2, depending of the host cell type and the time after infection. We also show an SsrB-independent, positive, direct regulation of *sseK1* by the two-component system PhoQ/PhoP and identify a PhoP box in the promoter region of SseK1.

## Materials and Methods

### Bacterial Strains, Bacteriophages and Strain Construction

*Escherichia coli* and* S. enterica* serovar Typhimurium strains used in this study are described in **Table 1**. *Salmonella* strains derive from the mouse-virulent strain ATCC 14028. Transductional crosses using phage P22 HT 105/1 *int201* (Schmieger, 1972) were used for strain construction (Maloy, 1990). To obtain phage-free isolates, transductants were purified by streaking on green plates. Green plates were prepared as described (Chan et al., 1972), except that methyl blue (Sigma) substituted for aniline blue. Phage sensitivity was tested by cross-streaking with the clear-plaque mutant P22 H5 (Chan et al., 1972).

**Table 1:** Bacterial strains and plasmids used in this study.

Strain/plasmid	Relevant characteristics	Source/reference
***Escherichia coli***
DH5α	*supE44 ΔlacU*169 (Ø80 *lacZΔ*M15) *hsdR17 recA1 endA1 gyrA96 thi-1 relA1*	Hanahan (1983)
XL1-Blue	*recA1 endA1 gyrA96 thi-1 hsdR17 supE44 relA1 Δlac-pro*/F’ *proAB lacI^q^ lacZ*ΔM15 Tn*10* (Tet^r^)	Bullock et al. (1987)
M15	*lac ara gal mtl*	Qiagen
***Salmonella enterica* serovar Typhimurium^**a**^**
14028	Wild-type	ATCC
55130	*phoQ24* (PhoP constitutive)	E. A. Groisman
SV4536	*Δdam-230*	Prieto et al. (2004)
SV4608	*trg::*Mu*d*J	Segura et al. (2004)
SV4699	*phoP7953::*Tn10, Tc^r^	Groisman et al. (1989), Segura et al. (2004)
SV4757	*rcsC54*	García-Calderón et al. (2005)
SV5049	*ΔrcsB*::Cm^r^	García-Calderón et al. (2007)
SV5373	*ΔhilA*	J. López-Garrido
SV5452	*ΔssrB*::Cm^r^	García-Calderón et al. (2007)
SV6017	*Δ*SPI2::Cm^r^	Baisón-Olmo et al. (2012)
SV6055	*Δ*SPI1::Km^r^	Baisón-Olmo et al. (2012)
SV6402	*ΔhilD*::Cm^r^	J. López-Garrido
SV7070	*ΔsseK1*::Km^r^	This study
SV7071	*sseK1::*3xFLAG, Km^r^	This study
SV7179	*ΔsseK1*	This study
SV7381	*sseK1::cyaA’,* Km^r^	This study
SV8165	*sseK1::lacZ* (translational fusion)	This study
**Plasmids**
pCE36	*aph* FRT *lacZY^+^* t*_his_* oriR6K	Ellermeier et al. (2002)
pCE40	*aph* FRT *‘lacZ lacY^+^* t*_his_* oriR6K	Ellermeier et al. (2002)
pCP20	*bla cat cI857* P_R_ *flp* pSC101 oriTS	Cherepanov and Wackernagel (1995)
pIC552	parent for *lacZ* transcriptional fusions, Ap^r^	Macián et al. (1994)
pIZ1673	pSIF003-R1 *ΔlacI*	Cardenal-Muñoz and Ramos-Morales (2011)
pIZ1949	pQE30-*phoP*	Cardenal-Muñoz and Ramos-Morales (2013)
pIZ1959	pIZ1673-SseK1(1-336)	This study
pIZ2095	pIC552- P*sseK1*(-500/+40)	This study
pIZ2112	pIC552- P*sseK1*(-500/+40)TT-73/-72CC	This study
pIZ2115	pSB377-P*sseK1*(-500/+40)	This study
pIZ2135	pSB377-P*sseK1*(-500/+40)TT-51/-50CC	This study
pIZ2136	pSB377-P*sseK1*(-500/+40)TT-62/-61CC	This study
pIZ2137	pSB377-P*sseK1*(-500/+40)TT-73/-72CC	This study
pIZ2154	pIC552- P*sseK1*(-500/+40)TT-62/-61, -73/-72CC	This study
pKD4	*bla* FRT *aph* FRT PS1 PS2 oriR6K	Datsenko and Wanner (2000)
pKD13	*bla* FRT *aph* FRT PS1 PS4 oriR6K	Datsenko and Wanner (2000)
pKD46	*bla* P_BAD_ *gam bet exo* pSC101 oriTS	Datsenko and Wanner (2000)
pREP4	*lacI*, Km^r^	Qiagen
pSB377	parent for *luxCDABE* transcriptional fusions, Ap^r^	Winson et al. (1998)

^a^Derivatives of these strains were used as indicated in the text.

### Bacterial Culture

The standard culture medium for *S. enterica* and *E. coli* was Luria-Bertani (LB) broth. For SPI1-inducing conditions, *S. enterica* strains were grown overnight at 37°C in LB-0.3 M NaCl medium without shaking. For SPI2-inducing conditions, bacteria were inoculated in low magnesium minimal medium (LPM) at pH 5.8, and incubated overnight at 37°C with shaking. LPM contained 80 mM 2-(*N*-morpholino) ethanesulfonic acid (pH 5.8), 5 mM KCl, 7.5 mM (NH_4_)_2_SO_4_, 0.5 mM K_2_SO_4_, 0.1% casamino acids, 38 mM glycerol, 337.5 μM K_2_HPO_4_-KH_2_PO_4_ (pH 7.4), and 8 μM MgCl_2_. For some experiments the concentration of NaCl, of MgCl_2_, or the pH of the medium were modified as indicated. Solid media contained 1.5% agar. Antibiotics were used at the following final concentrations in rich medium: kanamycin (Km), 50 μg ml^-1^; chloramphenicol (Cm), 20 μg ml^-1^; ampicillin (Ap), 100 μg ml^-1^; tetracycline (Tc), 20 μg ml^-1^. In minimal medium antibiotics were used at these concentrations: kanamycin, 125 μg ml^-1^; chloramphenicol, 5 μg ml^-1^; ampicillin, 50 μg ml^-1^; tetracycline, 10 μg ml^-1^. Plates for monitoring β-galactosidase activity contained 5-bromo-4-chloro-indolyl-β-D-galactopyranoside (X-Gal, final concentration, 40 μg ml^-1^). 10 mM sodium butyrate (Sigma) was added to the medium in some experiments.

### Mammalian Cell Culture

HeLa (human epithelial; ECAC no. 93021013), RAW264.7 (murine macrophages; ECACC no. 91062702), NRK-49F (normal rat kidney fibroblasts; ATCC CRL-1570), Cos-7 (monkey fibroblasts; ATCC CRL-1651), NIH3T3 (murine fibroblasts; ATCC CRL-1658), J774A.1 (murine macrophages; ATCC TIB-67) and Caco2 (human epithelial; ATCC HTB-37) cells were cultured in DMEM supplemented with 10% fetal calf serum and 2 mM L-glutamine. Sixty μg ml^-1^ penicillin, and 100 μg ml^-1^ streptomycin were included in the culture media (except for bacterial infection experiments). All cells were maintained in a 5% CO_2_ humidified atmosphere at 37°C.

### DNA Amplification with the Polymerase Chain Reaction and Sequencing

Amplification reactions were carried out in a T100 Thermal Cycler (Bio-Rad). The final volume of reactions was 50 μl, and the final concentration of MgCl_2_ was 1.5 mM. Reagents were used at the following concentrations: dNTPs, 300 μM; primers, 0.3 μM; and Taq polymerase (KAPA HiFi DNA Polymerase, Kapa Biosystems), 1 unit per reaction. The thermal program included the following steps: (i) initial denaturation, 3 min at 95°C; (ii) 25 cycles of denaturation (98°C, 20 s), annealing (60°C, 15 s), and extension (72°C, 30 s per kb); and (iii) final incubation at 72°C for 5 min, to complete extension. To generate directed mutations in the *sseK1* promoter cloned in pSB377 or pIC552 the thermal program included the following steps: (i) initial denaturation, 3 min at 95°C; (ii) 17 cycles of denaturation (98°C, 20 s), annealing (62°C, 15 s), and extension (72°C, 6 min); (iii) final extension at 72°C for 5 min. Primers are listed in **Table 2**. PCR constructs were sequenced with an automated DNA sequencer (Stab Vida, Oeiras, Portugal).

**Table 2:** Oligonucleotides used in this study.

Oligonucleotide/use	Sequence 5′-3′
***sseK1* deletion**
sseK1dP1	TAAAATATGTAATGAAGTAAGTATGGAGCA TTTAATTGTTGTGTAGGCTGGAGCTGCTTC
sseK1dP2	ATATTTTATGTATTCAATAGCATGATTATTGCCA TTTCCGCATATGAATATCCTCCTTAG
**Construction of *sseK1::lacZ* translational fusion**
sseK1P1b	CATGAACTTTGCGTAAACTGACTGGTATTCATT ATAATGTGTGTAGGCTGGAGCTGCTTC
sseK1P4	ATATGTTCCCGCGCTTTCAAAAAATGAATTGGTT AAAACTATTCCGGGGATCCGTCGACC
**Epitope tagging of SseK1**
sseK1P1flag	CAGTCAGTTTACGCAAAGTTCATGGGCGAGGCAT GTGCAGGACTACAAAGACCATGACGG
sseK1P2flag	ATATTTTATGTATTCAATAGCATGATTATTGC CATTTCCGCATATGAATATCCTCCTTAG
**Chromosomal *sseK1::cyaA’* fusion**
sseK1P1	CAGTCAGTTTACGCAAAGTTCATGGGCGAGGCA TGTGCAGCTGCAGCAATCGCATCAGGC
sseK1P2	ATATTTTATGTATTCAATAGCATGATTATTGCCAT TTCCGTTAGAAAAACTCATCGAGCATC
**Verification of chromosomal *sseK1::cyaA’* fusion**
sseK1E1	TTAATTGCTCACTGGCAGGG
sseK1E2	GCACTGCGATTTTAAAGTGG
cyaArev	CCTTGATGCCATCGAGTACG
**Construction of pIZ1959**
sseK1BampSIFfw	AGTCGGATCCAGGAGGAAATAT ATGATCCCACCATTAAATAG
sseK1BampSIFrev	GATCGGATCCACTGCACATG CCTCGCCCATG
**Construction of pIZ2095**
PsseK1-500fwBgl	AGTCAGATCTTTGGGACAATTACATTATG
PsseK1+40revXho	AGTCCTCGAGAACAATTAAATGCTCCATAC
**Construction of pIZ2115**
PsseK1-500fwEco	AGTCGAATTCTTGGGACAATTACATTATG
PsseK1+40revEco	AGTCGAATTCAACAATTAAATGCTCCATAC
**Construction of pIZ2135**
sseK1TT-51CCfw	GCTTAGTTTAGCATCTTCCAGCTGACAGCGATTGC
sseK1TT-51CCrev	GCAATCGCTGTCAGCTGGAAGATGCTAAACTAAGC
**Construction of pIZ2136**
sseK1TT-62CCfw	CCTCCGGTTAATGCTTAGCCTAGCATC TTTTAGCTGAC
sseK1TT-62CCrev	GTCAGCTAAAAGATGCTAGGCTAAGCATT AACCGGAGG
**Construction of pIZ2112 and pIZ2137**
sseK1TT-73CCfw	GTATTTATGTATCCTCCGGCCAATGCTTAGT TTAGCATC
sseK1TT-73CCrev	GATGCTAAACTAAGCATTGGCCGGAGGATAC ATAAATAC
**Construction of pIZ2154**
sseK1-73TT-62CCfw	CCTCCGGCCAATGCTTAGCCTAGCATCTTT TAGCTGAC
sseK1-73TT-62CCrev	GTCAGCTAAAAGATGCTAGGCTAAGCATTGG CCGGAGG
***sseK1 promoter***
FAMsseK1-500fw	TTGGGACAATTACATTATGTTTG
FAMsseK1+40rev	AACAATTAAATGCTCCATACTTAC
FAMsseK1-300fw	CTCGCCATTATAAAATACCTG
FAMsseK1-1rev	CATGATGATTATTAGCACATG
***slyB* promoter**
promslyBdir	AGACTTGCCTGTTGCGCAAC
promslyBrev	AAACGCTATTTCAGCATCCC
***phoN* promoter**
promphoNdir	AATGCGTGTCAGTCAGGCAC
promphoNrev	TTAGCTACGATCAGTGGTAG

### Plasmids

Plasmids used in this study are listed in **Table 1**. Plasmid pIZ2115 expressing a transcriptional *sseK1*::*lux* fusion was a derivative of pSB377 (a generous gift from P. Williams, University of Nottingham). To construct this plasmid, DNA from strain 14028 was used as a template for PCR amplification with the primers listed in **Table 2**. The amplified fragments were digested with *Eco*RI and ligated with *Eco*RI-digested pSB377. To generate point mutations in the *sseK1* promoter, pIZ2095 or pIZ2115 were used as templates for PCR amplification using primer pairs sseK1TT-51CCfw/sseK1TT-51CCrev, sseK1TT-62CCfw/sseK1TT-62CCrev, sseK1TT-73CCfw/sseK1 TT-73CCrev, or sseK1-73TT-62CCfw/sseK1-73TT-62CCrev. Products were digested with 1 μl of *Dpn*I (10 U μl^-1^) for 1 h at 37°C and used to transform *E. coli* DH5α. All constructs were confirmed by DNA sequencing.

### Generation of a *sseK1* Mutant

Disruption and replacement of *sseK1* with a Km resistance gene were performed as described previously (Datsenko and Wanner, 2000). Briefly, the Km resistance gene from plasmid pKD4 was PCR amplified with primers sseK1dP1 and sseK1dP2 (**Table 2**). The PCR product was used to transform the wild-type strain carrying the Red recombinase expression plasmid pKD46.

### Construction of *lacZ*, 3xFLAG, and *cyaA*’ Chromosomal Fusions

The Km resistance gene from plasmid pKD13 was PCR amplified with primers sseK1P1b and sseK1P4 (**Table 2**). The PCR product was used to transform the wild-type strain carrying the Red recombinase expression plasmid pKD46. The antibiotic resistance cassette introduced by the gene-targeting procedure described in the previous section was eliminated by recombination using the FLP helper plasmid pCP20 (Datsenko and Wanner, 2000). The FRT site generated by excision of the antibiotic resistance cassette was used to integrate plasmid pCE40 to generate a translational *lac* fusion (Ellermeier et al., 2002). Addition of a DNA fragment encoding the 3xFLAG epitope tag at the 3^′^ end of *sseK1* was carried out as described (Uzzau et al., 2001) using primers sseK1P1flag and sseK1P2flag. The protocol to generate a chromosomal *sseK1::cyaA’* translational fusion was recently described (Ramos-Morales et al., 2015).

### β-Galactosidase Assays

Levels of β-galactosidase were assayed as described (Miller, 1972), using the CHCl_3_/SDS permeabilization procedure. Bacteria were grown under SPI1 or SPI2-inducing conditions or modifications of these conditions as described in Section “Results.”

### Antibodies and Immunoblot

*Salmonella* strains were grown under different conditions. Usually, cultures in LB medium were diluted and grown in different media. The bacteria were then pelleted by centrifugation and resuspended in sodium dodecyl sulfate-polyacrylamide gel electrophoresis (SDS-PAGE) sample buffer. Proteins from the same numbers of bacteria were separated by gradient SDS-PAGE (Mini-PROTEAN TGX precast gels, 4–15%) and electrophoretically transferred to nitrocellulose filters for Western blot analysis using anti-Flag (M2) monoclonal antibodies (1:5000; Sigma), and anti-DnaK (8E2/2) monoclonal antibodies (1:5000; Assay Designs). Goat anti-mouse HRP-conjugated antibodies (1:5000; BioRad) and goat anti-rabbit HRP-conjugated antibodies (1:10000; GE Healthcare) were used as secondary antibodies. Intensities of SseK1-3xFLAG and DnaK bands were quantified using NIH ImageJ 1.42q software.

### Virulence Assays in Mice

Groups of three 8-weeks-old female BALB/c mice (Charles River Laboratories) were inoculated with a 1:1 ratio of two strains of *S. enterica* serovar Typhimurium: a *ΔsseK1*::Km^r^ null mutant and the wild-type (strain 14028). For oral inoculation, bacterial cultures were grown overnight at 37°C in LB without shaking. For intraperitoneal inoculation, bacteria were grown overnight at 37°C in LB with shaking, diluted into fresh medium (1:100), and grown to an OD_600_ of 0.3–0.6. Oral inoculation was performed by feeding the mice with 25 μl of 0.9% NaCl containing 0.1% lactose and 10^8^ CFU. Intraperitoneal inoculation was performed with 0.2 ml of 0.9% NaCl containing 10^5^ CFU. Bacteria were recovered from spleens 6 days (oral) or 2 days (intraperitoneal) after inoculation and colonies were enumerated on LB and LB with Km (to distinguish mutant and wild-type strains). A competitive index (CI) was calculated as the ratio between the *sseK1* mutant and the wild-type strain in the output (bacteria recovered from spleens) divided by their ratio in the input (initial inoculum). The experimental protocol was approved by the ethical committee of the University of Seville.

### Bacterial Infections of Cultured Cells

Mammalian cells were plated 24 h before infection in 24-well plates (Thermo Scientific) at 1.5 × 10^5^ cells per well, and incubated at 37°C with 5% CO_2_ in media without antibiotics. For infections under SPI1-inducing (invasive) conditions, bacteria grown overnight in LB-0.3 M NaCl in a tightly closed tube without shaking were added at a multiplicity of infection of 100. For infections of mammalian cells under non-invasive conditions, bacteria were grown in LB for 24 h at 37°C with shaking. Bacteria were centrifuged onto the cell monolayer at 200 g for 5 min and then incubated at 37°C with 5% CO_2_. The cell culture was washed twice with phosphate-buffered saline (PBS) 1 h post-infection (p.i.), overlaid with DMEM containing 100 μg ml^-1^ gentamicin, and incubated for 1 h. The culture was then washed twice with PBS, covered with DMEM with gentamicin 16 μg ml^-1^, and incubated for 2–14 h.

For invasion and proliferation assays, infections were carried out using a 10:1 mix of the *sseK1* mutant and a *trg*::Mu*d*J mutant (wild-type for invasion and intracellular proliferation but Lac^+^ due to the Mu*d*J insertion). CI for invasion and proliferation were calculated as previously described (Segura et al., 2004) after plating appropriate dilutions and enumerating white colonies (*sseK1*) and blue colonies (*trg*::Mu*d*J) in LB plates supplemented with 40 μg ml^-1^ 5-bromo-4-chloro-galactopyranoside (X-Gal). For invasion, the input was the initial mix of bacteria used in the infection and the output bacteria recovered 2 h p.i. For intracellular proliferation, bacteria were recovered 1.25 h p.i. (input) and 24 h p.i. (output).

### Bioluminescence Assays

Bacterial strains were grown under SPI1 or SPI2-inducing conditions. Samples of 150 μl were transferred into white, clear bottom, 96-well plates (Corning) and luminescence and OD_600_ were read using a Synergy HT microplate reader (BioTek). Conditions used for reading luminescence were: read type, endpoint; integration time, 1 s; emission, hole; position, top; sensitivity, 150. To measure luminescence of intracellular bacteria, RAW264.7 cells were plated into white, clear bottom, 96-well plates at 3 × 10^4^ cells per well, and were infected 24 h later with non-invasive bacteria, according to the protocol described in Section “Bacterial Infections of Cultured Cells.” Luminescence was measured 2, 4, and 8 h p.i. and the numbers of CFU per well were calculated after incubation with 1% Triton X-100 in PBS for 10 min at 37°C to release bacteria, plating appropriate dilutions in LB with Ap, and counting colonies after 24 h of incubation at 37°C.

### Protein Translocation Assay

Following the infections described above, the translocation of the SseK1-CyaA’ fusion into the eukaryotic cells was monitored by measuring the levels of cAMP. The infected cells were lysed and the level of cAMP in the lysates was determined using a colorimetric direct cAMP enzyme immunoassay kit (Arbor Assays) according to the manufacturer’s instructions.

### Protein Purification and Phosphorylation

His_6_-PhoP protein was produced and purified as previously described (Gal-Mor et al., 2011) with some modifications (Cardenal-Muñoz and Ramos-Morales, 2013). For binding assays, *S. enterica* His_6_-PhoP was phosphorylated with acetyl phosphate as previously described (Tang et al., 2012) with modifications. Briefly, His_6_-PhoP was incubated in 20 μl of phosphorylation buffer (50 mM Tris-HCl pH 7.5, 50 mM KCl, 10 mM MgCl_2_) containing 10 mM acetyl phosphate (Sigma-Aldrich) for 1 h at 37°C.

### Electrophoretic Mobility Shift Assay (EMSA)

DNA fragments used for the PhoP binding assay were amplified by PCR using *Salmonella* 14028 as a template. The primers, listed in **Table 2**, were labeled with 6-carboxyfluorescein (FAM). PCR amplification rendered fragments of 281, 355, and 540 (or 300) bp for *phoN*, *slyB,* and *sseK1* promoters, respectively. The binding assay was carried out as previously described (Tang et al., 2012) with modifications. Briefly, a solution of 5 nM of FAM-labeled DNA and 0, 0.125, 0.25, 0.5, 1, and 2 μM of phosphorylated His_6_-PhoP was prepared in binding buffer (50 mM Tris-HCl pH 8.0, 50 mM KCl) in a total volume of 20 μl and incubated for 30 min at room temperature. Protein-DNA complexes were subjected to electrophoresis at 4°C in a 6% non-denaturing acrylamide:bisacrylamide (29:1) gel in 0.5X Tris-borate-EDTA buffer. Images were acquired using a Fujifilm FLA-5100 system.

### Statistical Analysis

Student’s *t*-test was used to analyze differences in β-galactosidase activities and light emission. This test was also used to analyze every CI against the null hypothesis that the mean is not significantly different from 1. *P* values of 0.05 or less were considered significant.

## Results

### Contribution of SseK1 to Virulence in Mice

**FIGURE 1:**
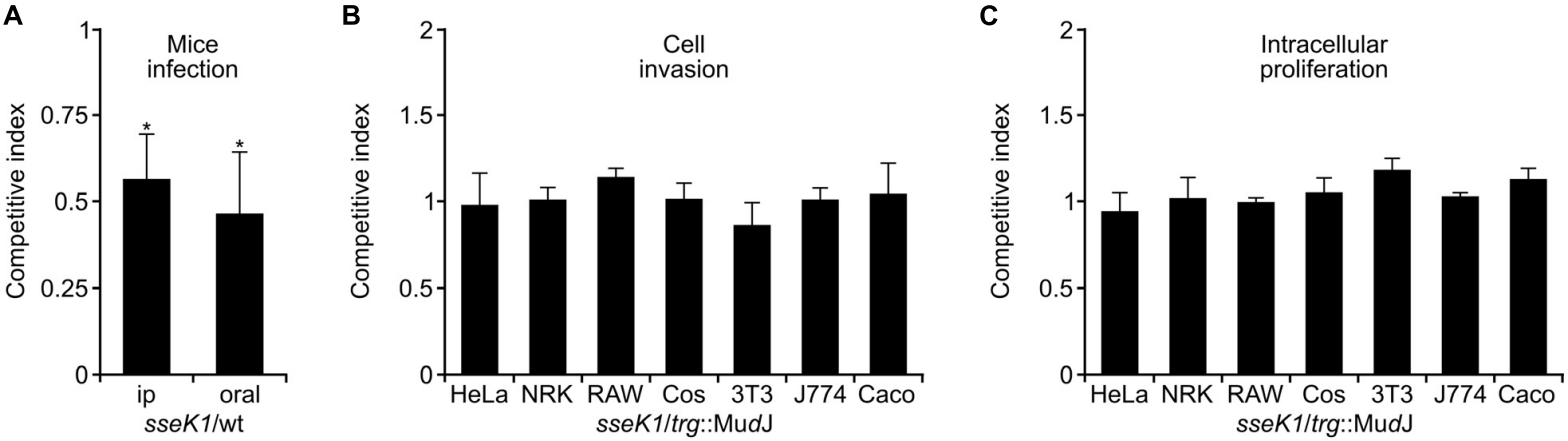
**Competitive index (CI) analysis for an *sseK1* null mutant. (A)** Graphical representation of CI analysis of a strain carrying a mutation in *sseK1* after intraperitoneal (ip) and oral mice infections. **(B)** Analysis of invasion of the *sseK1* mutant in mixed infections with a *trg*::Mu*d*J mutant used as the wild-type strain. **(C)** Analysis of intracellular proliferation of the *sseK1* mutant in mixed infections with a *trg*::Mu*d*J mutant used as the control strain. The CIs are the means from three infections. Error bars represent the SD. wt, wild-type strain. Asterisks denote that the CIs are significantly different from 1 for a *t*-test *P* value < 0.05.

The role of SseK1 and SseK2 in virulence was previously evaluated by infecting BALB/c mice with *sseK1, sseK2* or* sseK1 sseK2* mutants, but no attenuation was detected using a time to death assay after intraperitoneal infections (Kujat Choy et al., 2004). More recently, a sensitive method, the CI, revealed significant attenuation of the *sseK1 sseK2 sseK3* triple mutant and of the *sseK1 sseK2* double mutant, but no attenuation of the *sseK3* single mutant, after oral infections (Brown et al., 2011). These results prompted us to analyze the specific contribution of SseK1 to *Salmonella* virulence. An *sseK1* null mutant was generated and the CI for this single mutant compared to the wild-type strain (*S. enterica* serovar Typhimurium strain 14028). Significant attenuation (*P* < 0.05) was observed both after intraperitoneal and after oral infections of BALB/c mice (**Figure 1A**). Specific contribution of SseK1 to invasion and intracellular proliferation was assessed calculating the CI of the *sseK1* mutant against the *trg*::Mu*d*J strain [wild-type for invasion and intracellular proliferation (Segura et al., 2004)] in a variety of mammalian cell types: HeLa (human epithelial), NRK-49F (rat fibroblasts), RAW264.7 (murine macrophages), Cos-7 (monkey fibroblasts), 3T3 (murine fibroblasts), J774.A1 (murine macrophages), and Caco2 (human epithelial). No significant defect was detected for this mutant (*P* > 0.05; **Figures 1B,C**). Together, the results shown in this section suggest that SseK1 is necessary for full virulence of *Salmonella* in mice but that it does not contribute specifically to invasion or intracellular proliferation, at least in the cell types and under the conditions tested.

### Synthesis and Translocation into Mammalian Cells of SseK1 Under SPI1 and SPI2 Inducing Conditions

**FIGURE 2:**
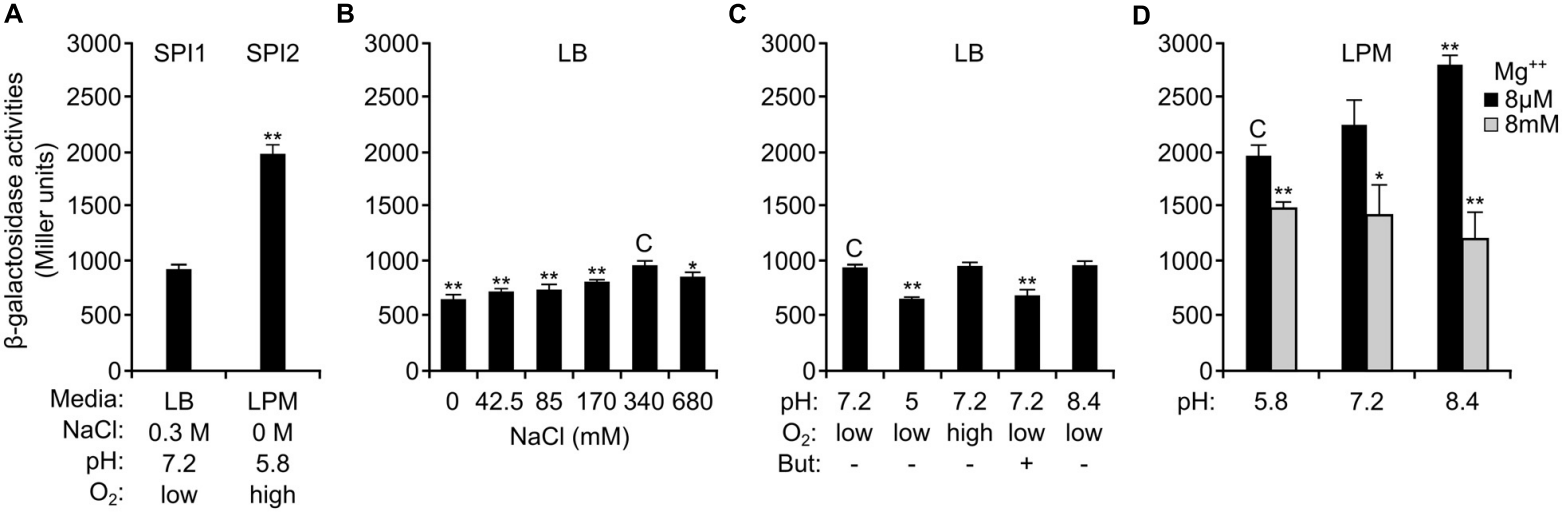
**Expression of *sseK1* in different culture media.** β-Galactosidase activities were measured from overnight cultures of a *Salmonella enterica* strain carrying a chromosomal *sseK1::lacZ* translational fusion. **(A)** Bacteria were incubated overnight at 37°C in LB with 0.3 M NaCl without shaking (SPI1) or in LPM with shaking (SPI2). **(B)** Different concentrations of NaCl were used to test the role of osmolarity on *sseK1* expression under SPI1-inducing conditions.** (C)** The effects of pH, oxygen limitation and butyrate on *sseK1* expression were tested in LB. **(D)** Activities were measured after growth in LPM with different pH and Mg^++^ concentrations, as indicated. Means and SD from three independent β-galactosidase measurements are shown. Statistical significance is shown by asterisks representing *t*-test *P* values: ^∗^*P* < 0.05; ^∗∗^*P* < 0.01. C: reference for statistical comparison.

Although expression and secretion to culture media of SseK1 was detected under SPI1 and SPI2-inducing conditions, this *Salmonella* effector was described as translocated into human epithelial HeLa cells specifically through the T3SS2 (Kujat Choy et al., 2004). These previous results were obtained based on SseK1-2HA and SseK1-CyaA’ fusions expressed from a plasmid. To carry out a more detailed analysis of the expression of *sseK1,* we constructed a chromosomal *lacZ* translational fusion in the native *sseK1* locus. This fusion permits quantification of the physiological levels of expression of this gene by measuring β-galactosidase activities (see Materials and Methods). As seen in **Figure 2A**, *sseK1* was expressed under SPI1-inducing conditions (LB, 0.3 M NaCl, without aeration) but its expression was significantly higher (*P* < 0.01) under SPI2-inducing conditions (LPM, pH 5.8, high aeration). Variants of these conditions were tested to detect relevant factors influencing the expression of *sseK1*. Changes in osmolarity in the SPI1-inducing medium had little but significant impact (*P* < 0.01 or 0.05; **Figure 2B**), and the maximum expression in this medium was obtained with the original NaCl concentration (0.3 M). Hypoxia did not appear to be an important factor in the expression of *sseK1* but butyrate, a fermentation product found in the intestine that is known to downregulate SPI1 genes, caused a significant repression (*P* < 0.01) of *sseK1* expression (**Figure 2C**). Interestingly, although the low Mg^++^ concentration that is present in the medium used to induce SPI2 (LPM) was a factor contributing to expression of *sseK1*, the acidic pH of the same medium had a negative impact (**Figure 2D**) and the highest expression was observed in LPM with low Mg^++^ concentration at pH 8.4 (*P* < 0.01). A general conclusion of these experiments is that expression of *sseK1* is not restricted to SPI1- or SPI2-inducing conditions resulting in coexpression of this gene with both islands.

**FIGURE 3:**
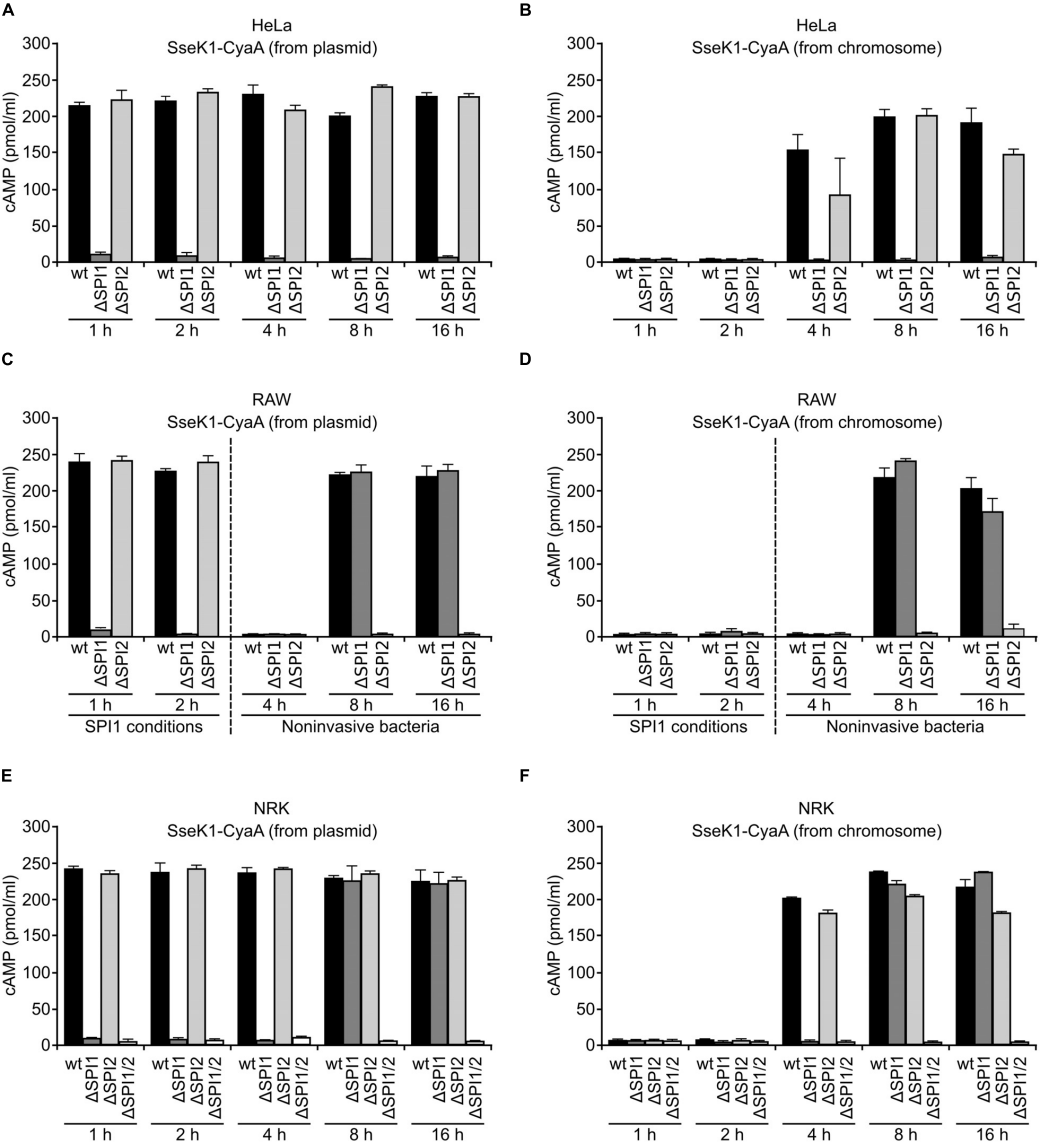
**Translocation of SseK1 into mammalian cells.** Human epithelial HeLa cells **(A,B)**, RAW264.7 murine macrophage-like cells **(C,D)** and NRK-49F normal rat kidney fibroblasts **(E,F)** were infected with derivatives of *S. enterica* serovar Typhimurium 14028 (wild-type, wt, ΔSPI1, ΔSPI2 and ΔSPI1 ΔSPI2 strains) carrying a plasmid expressing an SseK1-CyaA’ fusion from a constitutive promoter **(A,C,E)** or a chromosomal SseK1-CyaA’ fusion expressed under the native *sseK1* promoter **(B,D,F)**. Bacteria were grown under SPI1-inducing conditions for most infections. Non-invasive bacteria were used specifically for infections of RAW264.7 cells for 4, 8, and 16 h. To detect translocation, the level of cAMP was measured 1, 2, 4, 8, and 16 h p.i. Means and SD from triplicate experiments are represented.

The results shown above are compatible with translocation of SseK1 through T3SS1 and T3SS2. A detailed analysis of this possibility was carried out using two kinds of SseK1-CyaA’ fusions: the first one was prepared in a plasmid and its expression was driven by a constitutive promoter; the second one was generated in the chromosome under the control of the native promoter. In both cases the whole SseK1 protein was fused to the catalytic domain of the calmodulin-dependent adenylate cyclase from *Bordetella pertussis*. *Salmonella* strains (wild-type and mutants lacking T3SSs) expressing these fusions were used to infect three mammalian cell types: human epithelial HeLa cells, murine RAW264.7 macrophages and rat NRK-49F fibroblasts. Translocation of the fusion into host cells was tested 1, 2, 4, 8, and 16 h p.i. and was detected as an increase in cAMP concentration in the cell culture (**Figure 3**). All the infections were carried out using invasive bacteria (grown under SPI1-inducing conditions) except long infections (4–16 h) of RAW cells to prevent rapid macrophage pyroptosis induced by invasive bacteria (Fink and Cookson, 2007). Interestingly, different patterns of translocation were observed depending on the host cell and on the fusion. The main conclusions are: (i) Translocation of SseK1 at short times (1 and 2 h p.i.) is only observed when the fusion is constitutively expressed from a plasmid (**Figures 3A,C,E**) and is dependent on T3SS1 in the three cell types. (ii) When *sseK1* is expressed from its own promoter (**Figures 3B,D,F**), translocation of SseK1 starts at 4 h p.i. in epithelial cells and fibroblasts, and at 8 h p.i. in macrophages. Under these conditions, translocation into HeLa cells depends on T3SS1, translocation into RAW cells depends on T3SS2, and translocation into NRK cells occurs through both systems.

### SsrB Independent Regulation of *sseK1* by PhoQ/PhoP

**FIGURE 4:**
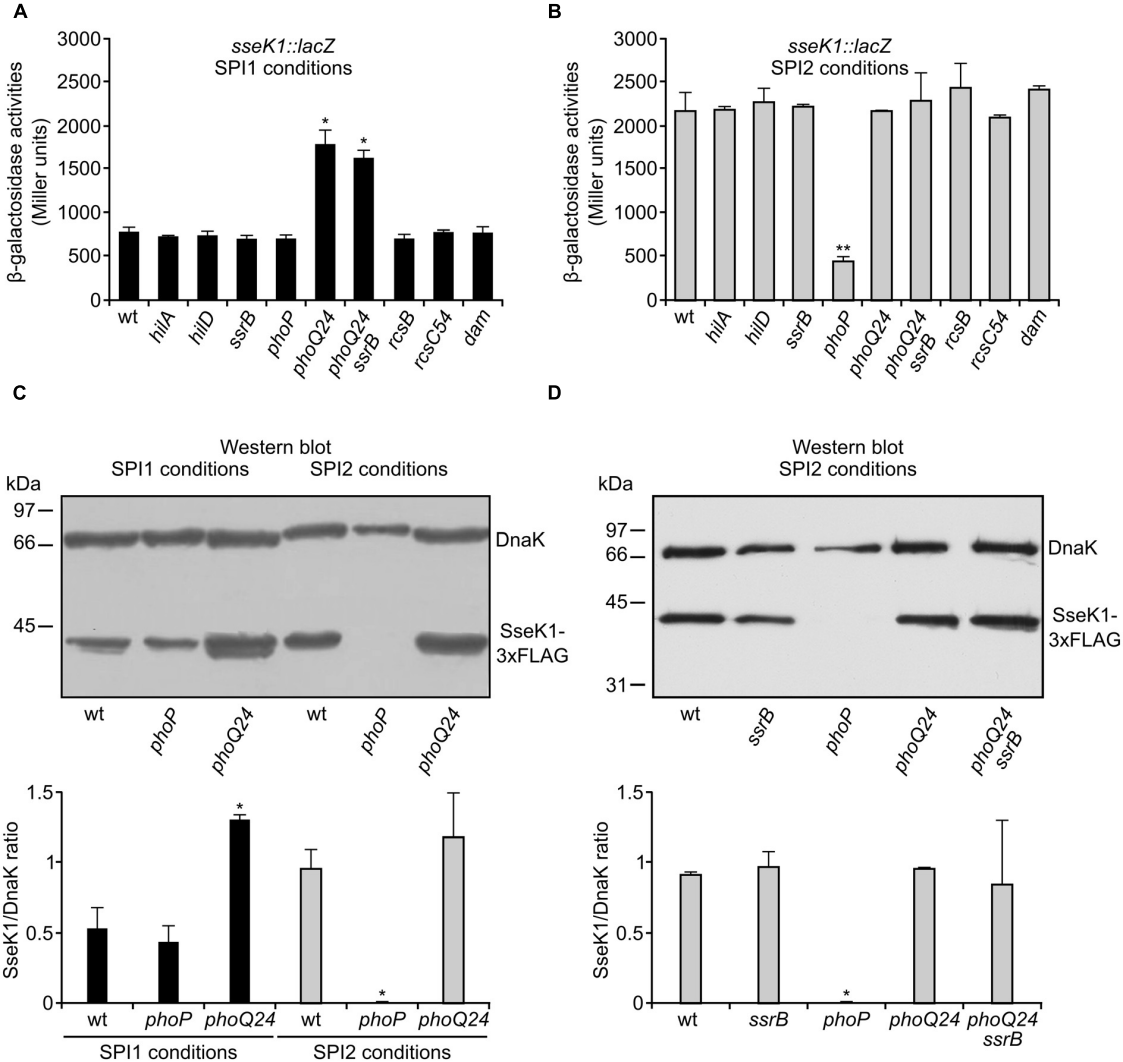
**Positive regulation of the expression of *sseK1* by the PhoQ/PhoP system.** β-Galactosidase activities were measured from SPI1-inducing **(A)** and SPI2-inducing **(B)** cultures of several *S. enterica* serovar Typhimurium strains: wild-type 14028 (wt), null mutants (*hilA, hilD, ssrB, rcsB*, and *dam*), and mutants with constitutive activation of the PhoQ/PhoP system and the Rcs system, respectively (*phoQ24* and *rcsC54*), carrying an *sseK1::lacZ* translational fusion. The role of SsrB in the regulation by PhoP was investigated in a double mutant *phoQ24 ssrB*. Means and SD from three independent β-galactosidase measurements are shown. The effect of the PhoQ/PhoP system **(C)** and of SsrB **(D)** on *sseK1* expression at the protein level was assessed by immunoblot analysis using strains expressing SseK1-3xFLAG. A monoclonal anti-FLAG antibody was used to detect the fusion protein and a polyclonal anti-DnaK antibody was used to get a loading control. Representative gels are shown together with quantification of bands (SseK1/DnaK ratio) from duplicate gels. Statistical significance of the differences between wt and mutant strains is shown by asterisks representing *t*-test *P* values: ^∗^*P* < 0.05; ^∗∗^*P* < 0.01.

We took advantage of the chromosomal *sseK1::lacZ* fusion to look for genetic factors controlling *sseK1* expression. We tested the effect of mutations in genes encoding important virulence regulators: HilA, HilD, SsrB, PhoP, RcsB, and Dam. HilA and HilD are positive regulators of SPI1 (Bajaj et al., 1995; Schechter and Lee, 2001). SsrB is the main positive regulator of the expression of SPI2 (Cirillo et al., 1998). Both islands are regulated by PhoP, that positively regulates SPI2 through SsrB and negatively regulates SPI1 through HilA (Bajaj et al., 1996; Bijlsma and Groisman, 2005). RcsB represses SPI1 through HilD (Mouslim et al., 2004; Lin et al., 2008). Dam is an adenine methylase that activates SPI1 through HilD (López-Garrido and Casadesús, 2010). In addition to null mutations in all these genes, the point mutations *phoQ24* and *rcsC54* were also used. These mutations result in constitutive activation of the two-component system PhoQ/PhoP and of the phosphorelay system RcsC/RcsD/RcsB, respectively. The level of expression of *sseK1::lacZ* was measured in liquid bacterial cultures grown under SPI1 (**Figure 4A**) or SPI2 (**Figure 4B**) inducing conditions. The results suggest that PhoP is a positive regulator of *sseK1* expression since a significant decrease in expression was observed in a *phoP*-null mutant under SPI2 inducing conditions (*P* < 0.01). This conclusion is confirmed by the positive effect of the activating mutation *phoQ24* on *sseK1* expression under SPI1-inducing conditions (*P* < 0.05). We also assessed the effect of the *phoP* mutation on SseK1 at the protein level using a chromosomal 3xFLAG fusion (**Figure 4C**). The effect was more dramatic at this level suggesting some kind of postranslational regulation in addition to the modulation of transcription that is expected for PhoP. No effect was detected for mutations affecting the other regulators that were tested in these assays, including SsrB. Lack of SsrB had no significant impact on *sseK1* expression even in a *phoQ24* background (**Figure 4D**), providing evidence for SsrB-independent upregulation of *sseK1* by the PhoQ/PhoP system.

### Direct Regulation of *sseK1* Expression by PhoP

**FIGURE 5:**
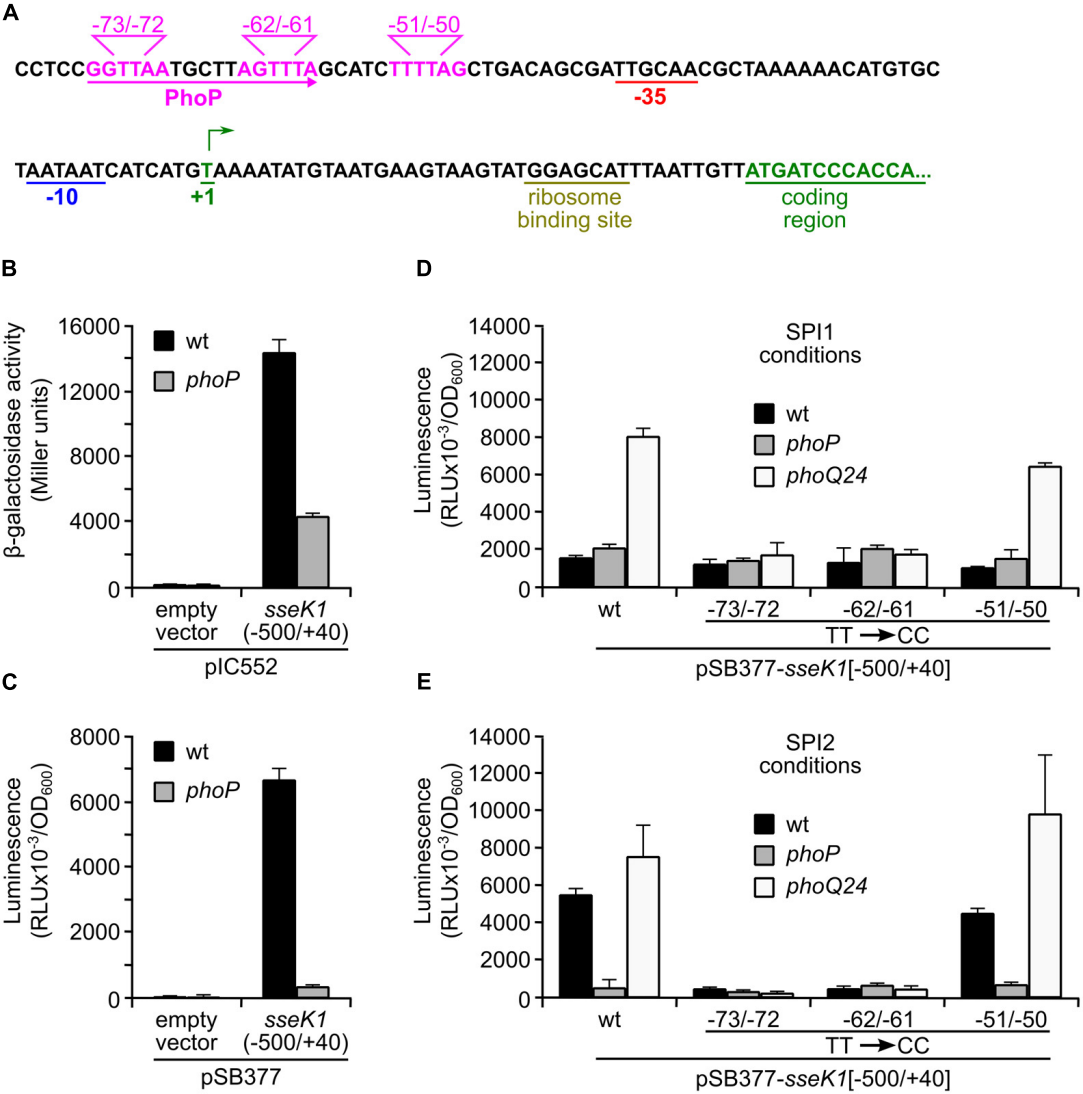
**Identification of a PhoP box in the promoter of *sseK1*. (A)** Analysis of the promoter region of *sseK1*. The sequence surrounding the transcriptional start site (+1) is shown. The start of the coding sequence, the putative ribosomal binding site and the consensus sequences for σ^70^-dependent transcription (-10 and -35) are indicated. Putative PhoP-binding motifs are marked in pink and a putative PhoP-box is underlined with an arrow. A fragment of DNA containing the promoter region and 5^′^ untranslated region of *sseK1* (-500/+40) was inserted into plasmid pIC552 to generate a *lacZ* transcriptional fusion **(B)** and into plasmid pSB377 to generate a *luxCDABE* transcriptional fusion **(C)**. These plasmids and the corresponding original empty vectors were introduced into *S. enterica* serovar Typhimurium strain 14028 (wt) or a *phoP*-null mutant, and β-galactosidase activities or luminescence, respectively, were measured in cultures grown to stationary phase in liquid LPM at pH 5.8. Luminescence was also measured from the wt, *phoP* and *phoQ24* strains carrying derivatives of pSB377 with the promoter region of *sseK1* or variants with the indicated mutations and grown under SPI1 **(D)** or SPI2 **(E)** inducing conditions. RLU: relative light units.

Next, we reasoned that since PhoP regulates *sseK1* in an SsrB-independent manner, it could be a direct regulator of this gene. To test this possibility, we decided to analyze the promoter region of *sseK1* looking for a putative PhoP-binding site. According to a previous global analysis carried out on *S. enterica* serovar Typhimurium strain SL1344 (Kroger et al., 2012), the transcriptional start site of *sseK1* is a T located 40 nucleotides upstream of the translational start codon. Visual inspection revealed the presence of putative -10 and -35 consensus motifs for σ70-dependent transcription with the appropriate spacing (**Figure 5A**). In addition, a sequence resembling the PhoP box consensus motif (T/G)GTTTA-NNNNN-(T/G)GTTTA (where N is any nucleotide), was found at position -75/-59 relative to the transcriptional start site. Another putative half box, less similar to the expected consensus, was located at -53/-48. The role of this region in driving the transcription of *sseK1* was tested using two different promoter probe plasmids: pIC552 and pSB377. A DNA fragment containing the promoter and 5^′^ untranslated regions of *sseK1*, from -500 to +40, was cloned into these plasmids to generate a *lacZ* transcriptional fusion (**Figure 5B**) and a bioluminescent *lux* transcriptional fusion (**Figure 5C**), respectively. Expression of the fusions in wt and *phoP* backgrounds indicated that the cloned region contained the signals necessary for expression and PhoP-mediated regulation of *sseK1*. To test the relevance of the putative PhoP binding sites, three independent mutants were obtained in the *lux* fusion plasmid. Each mutant was constructed by exchanging the conserved TT motif in the middle of a putative half PhoP box sequence for CC (**Figure 5A**). Whereas mutations at positions -51/-50 had no effect on the expression of the fusion, alteration of the -62/-61 or the -73/-72 motifs completely abrogated PhoP regulation of the fusion under SPI1 (**Figure 5D**) and SPI2-inducing conditions (**Figure 5E**). These results suggest that the region -75/-59 constitutes a complete PhoP binding box.

**FIGURE 6:**
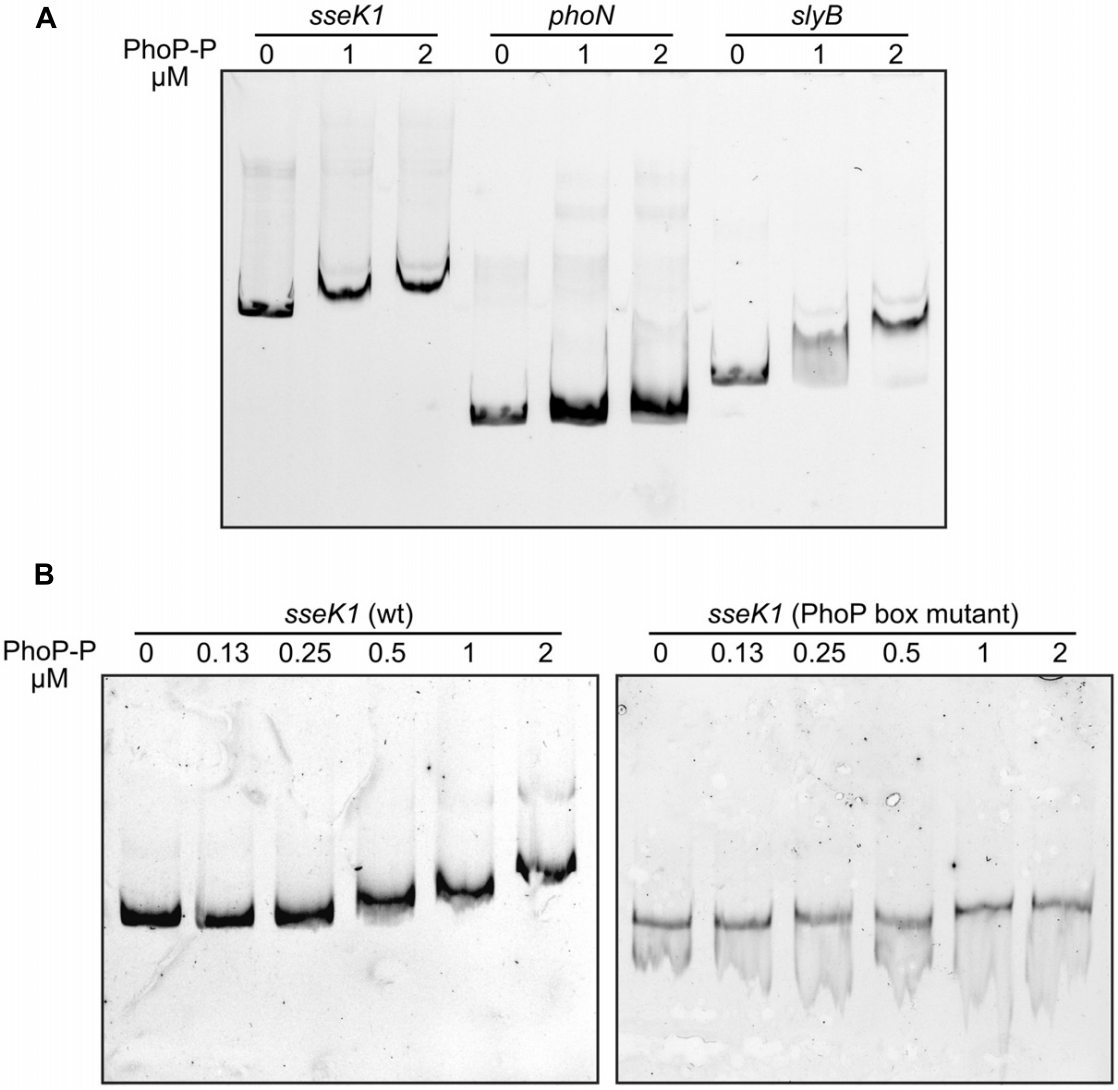
**Direct interaction of phosphorylated PhoP with the promoter region of *sseK1*.** Purified His_6_-PhoP was phosphorylated *in vitro* with acetyl phosphate. **(A)** DNA fragments containing the promoter regions of *sseK1* (-500/+40), *phoN* and *slyB* were PCR amplified using fluorochrome-labeled primers and incubated with the indicated concentrations of phosphorylated His_6_-PhoP (PhoP-P). Electrophoretic mobility shift assays were used to detect binding. **(B)** DNA fragments containing the promoter regions of *sseK1* (-300/-1) wild-type (wt) or with mutations T– > C at positions -73, -72, -62 y -61 (PhoP box mutant) were PCR amplified using fluorochrome-labeled primers and incubated with the indicated concentrations of phosphorylated His_6_-PhoP (PhoP-P). Electrophoretic mobility shift assays were used to detect binding.

Finally, an electrophoretic mobility shift assay was used to analyze the binding of PhoP to the promoter of *sseK1*. The promoters of *slyB* and *phoN* were used as positive and negative controls, respectively. Phosphorylated His_6_-PhoP and PCR-amplified DNA fragment containing the relevant promoters were used in these experiments. As seen in **Figure 6A**, PhoP was able to bind to the *slyB* and *sseK1* promoters and no binding was observed to the *phoN* promoter. In addition, mutations at positions -61/-62 and -72/-73, prevented PhoP binding (**Figure 6B**). These results provide additional support to the hypothesis that the region -75/-59 is a PhoP binding box.

### Expression of *sseK1* Inside Macrophages

**FIGURE 7:**
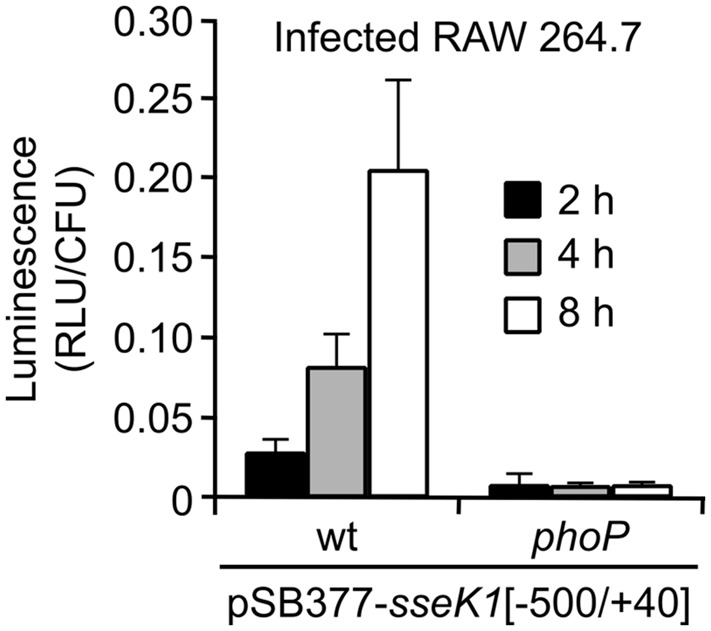
**Intracellular PhoP-dependent expression of *sseK1*.** Two strains of *S. enterica* serovar Typhimurium (wild-type, wt, and *phoP* mutant) carrying a plasmid expressing an *sseK1::luxCDABE* transcriptional fusion (pSB377-*sseK1*[-500/+40]) were grown for 24 h in LB at 37°C with aeration (non-invasive conditions). These bacteria were used to infect RAW264.7 murine macrophage-like cells and luminescence produced by intracellular bacteria was measured 2, 4, and 8 h p.i.

We took advantage of the *sseK1::lux* transcriptional fusion described above to study expression of *sseK1* during infection of RAW264.7 macrophages. *Salmonella* strains expressing this fusion were used to infect cultures of these cells in 96-well plates. As seen in **Figure 7**, luminescence per wild-type CFU was increased 4 and 8 h p.i. compared to 2 h p.i., suggesting that *sseK1* expression is induced, together with T3SS2, in response to intravacuolar signals. In contrast, intracellular induction was not observed in a *phoP* null mutant, giving additional support to the conclusion that PhoP is a positive regulator of the expression of *sseK1*.

## Discussion

Although the functions of the members of the SseK family of T3SS effectors are unknown, their amino acid sequence similarities suggested some redundancy in their functions. In previous studies, virulence attenuation was only shown for the triple *sseK1 sseK2 sseK3* or the double *sseK1 sseK2* mutants. The results presented here, however, clearly show that SseK1 is important in itself during the systemic phase of the infection in the mouse model, since a single *sseK1* mutant is significantly attenuated after oral and intraperitoneal infections (**Figure 1**). In addition, our efforts to detect secretion of SseK2 and SseK3 were unsuccessful, probably due to the low expression levels of these proteins under the conditions tested (data not shown). Our results also suggest that SseK1 is not necessary for invasion or intracellular proliferation in seven different mammalian cell lines. This is in agreement with previous attempts that were unable to find a phenotype following infection of HeLa, Caco2 or RAW264.7 cells (Kujat Choy et al., 2004; Brown et al., 2011; Buckner et al., 2011). However, one of these reports showed a 60% reduction in the replication index of the triple mutant *sseK1 sseK2 sseK3* inside RAW264.7 macrophages (Buckner et al., 2011). The discrepancy between results obtained in different laboratories may be a consequence of differences in experimental details including specific *Salmonella* strains used and multiplicity of infection.

SseK1 was initially described as a T3SS2 effector in HeLa cells (Kujat Choy et al., 2004; Brown et al., 2011). Here, we carried out a detailed analysis of translocation of this effector using three host cell lines, from three different mammalian species, and two different CyaA’ fusions (**Figure 3**). Our results suggest several conclusions and comments: (i) A first general conclusion is that SseK1 can be secreted through T3SS1 and T3SS2, although with different kinetics depending on the host cell type. Hence, the examination of as many host cell types as possible is essential to fully understand the function of T3SS effectors. (ii) Translocation at short time p.i. (1–2 h) was T3SS1-dependent but was only observed when the SseK1-CyaA’ fusion was expressed from a constitutive promoter in a plasmid. This result suggests that under physiological conditions (expression from its own promoter in the chromosome) SseK1 is not synthetized at sufficient level to allow detectable translocation before invasion of the host cell. It also indicates that the use of chromosomal fusions is more reliable in order to get conclusions about the conditions necessary for translocation of T3SS effectors. (iii) Translocation into epithelial HeLa cells was dependent on T3SS1, since it was not detected in a mutant lacking this system (**Figure 3B**). This result may be partially explained by the fact that the trigger mechanism mediated by T3SS1 is necessary for the invasion of these cells. As a consequence, translocation from internalized bacteria through T3SS2, if it existed, would not be detected using the ΔSPI1 mutant. (iv) Translocation into NRK fibroblasts, although T3SS1-dependent at 4 h p.i., appears to occur through both systems at 8 and 16 h p.i., since simultaneous inactivation of T3SS1 and T3SS2 is necessary to abolish the increase in cAMP (**Figure 3F**). Detection of T3SS2-dependent translocation using the T3SS1 mutant is possible in this model because invasion of fibroblasts can take place using a multiplicity of entry mechanisms (Aiastui et al., 2010). (v) Translocation of SseK1 into macrophages infected with non-invasive bacteria was T3SS2-dependent and was detected 8 and 16 h p.i., but not 4 h p.i. (**Figure 3D**). This is similar to the timing observed for T3SS2-dependent SseK1 translocation into fibroblasts infected with invasive bacteria and suggests that this system begins to be functional between 4 and 8 h after internalization in both cell types. The results obtained in RAW cells also suggest that non-invasive phagocytized bacteria are unable to induce T3SS1 inside these cells.

These results fit well into the context of a previous report showing simultaneous expression of T3SS1 and T3SS2 inside HeLa cells (Hautefort et al., 2008). This initially surprising coexpression was explained by the existence of two subpopulations of *Salmonella* in epithelial cells: a T3SS2-induced intravacuolar subpopulation and a T3SS1-induced cytosolic subpopulation (Knodler et al., 2010). Cytosolic *Salmonella* are also detected in fibroblasts and macrophages, although the permissiveness for *Salmonella* survival and replication in the cytosol is dependent upon the cell type (reviewed in Knodler, 2015).

In this study, we also used a combination of *lac*, 3xFLAG and *lux* fusions to analyze the environmental conditions and the genetic factors involved in the regulation of the expression of *sseK1*. Maximal expression in rich medium was obtained with 0.3 M NaCl and modest repression was observed with lower and higher salt concentrations (**Figure 2B**). Two additional factors that decreased expression of *sseK1* in rich medium were low pH and butyric acid (**Figure 2C**), a major short chain fatty acid produced in the intestine by anaerobic bacterial fermentation. This organic acid is known to repress SPI1 and other T3SS1-related genes (Lawhon et al., 2002; Gantois et al., 2006; Gong et al., 2009; Cardenal-Muñoz and Ramos-Morales, 2011). Expression in a minimal medium mimicking intravacuolar conditions (LPM) was higher than in rich medium (**Figure 2A**). However, acidic pH, which is one of the environmental cues used to induce SPI2, had a negative impact on the expression of *sseK1* also in minimal medium (**Figure 2D**). These data, together with translocation data shown in **Figure 3** and discussed above, suggest that expression of *sseK1* could be partially repressed during passage through the stomach and the gut, but it would be induced after invasion of host cells and specially after release into the cytosol of non-phagocytic cells.

Among the SPI1 and SPI2 regulators that we tested, only the PhoQ/PhoP two-component regulatory system had a significant effect on the expression of *sseK1* (**Figure 4**). PhoQ is a membrane protein that activates PhoP in response to low Mg^++^ concentration (García Véscovi et al., 1996; Montagne et al., 2001). PhoP is a transcription factor that regulates expression of about 3% of the *Salmonella* genes (Miller and Mekalanos, 1990). These genes are involved in the control of physiological and virulence functions. Positive regulation of *sseK1* by this system is consistent with the effect of Mg^++^ concentrations on its expression (**Figure 2D**).

The results obtained at the protein level with an SseK1-3xFLAG fusion are consistent with a previous report using a 2xHA fusion (Kujat Choy et al., 2004) and indicated that SseK1 was undetectable in a *phoP* null background. Our results also showed that this dramatic effect is observed specifically under SPI2-inducing conditions (**Figure 4C**), suggesting that under SPI1-inducing conditions there are other unidentified factors allowing synthesis of SseK1 in the absence of PhoP. The comparison between the results obtained at the protein level (Western blot in **Figures 4C,D**) and the results obtained using a chromosomal *sseK1::lacZ* translational fusion (β-galactosidase activities in **Figure 4B**) also suggests an indirect posttranslational effect in addition to the transcriptional effect that is expected for a regulator like PhoP.

Whereas PhoQ/PhoP is considered an ancestral regulatory system that is conserved in enteric bacteria and senses Mg^++^ concentrations, the two-component system SsrA/SsrB is *Salmonella*-specific and is activated by acidic pH (Miao et al., 2002; Mulder et al., 2015). SsrB is necessary for the expression of T3SS2 and some of its effectors that are encoded outside SPI2 (Ochman et al., 1996; Cirillo et al., 1998; Hensel et al., 1998; Worley et al., 2000). Since PhoP controls expression of the response regulator SsrB at the transcriptional level and of the sensor SsrA at a posttranscriptional level (Bijlsma and Groisman, 2005), it also indirectly regulates expression of SsrB-regulated genes. However, our epistasis analysis combining an *ssrB* null mutation with a *phoQ24* activating mutation (**Figure 4**) clearly showed that the effect of PhoP on *sseK1* was SsrB-independent, which is consistent with the induction of *sseK1* expression by low Mg^++^ concentrations but not by low pH. This result also suggested the possibility of direct regulation of *sseK1* by PhoP. Two lines of evidence support this hypothesis: (i) PhoP regulation of a *lux* transcriptional fusion was abrogated by mutations in a putative PhoP-box that was detected in the promoter region of *sseK1* (**Figure 5**). (ii) Binding of PhoP to the promoter region of *sseK1* was confirmed by EMSA analysis (**Figure 6A**). In addition, mutation of the putative PhoP-box significantly reduced binding (**Figure 6B**).

The bioluminescent fusion used here showed great sensitivity and was used to demonstrate *in vivo* PhoP-dependent induction of *sseK1* inside macrophages (**Figure 7**), giving stronger support to the conclusions obtained using a culture medium (LPM) that imitates intravacuolar conditions (**Figure 4**). This fusion could also be, in principle, useful for future experiments regarding the study of expression of *sseK1* inside animal models.

In summary, our results suggest that the T3SS effector SseK1 is a virulent factor that responds to a complex array of environmental signals. Expression of *sseK1* is directly activated by PhoP under SPI2-inducing conditions and, probably, by other unknown regulators under SPI1-inducing conditions. In response to these signals and regulators SseK1 is expressed and translocated through both T3SS1 and T3SS2 when *Salmonella* is inside the host cell. Additional experiments will be necessary to understand the specific role of SseK1 during infections.

## Conflict of Interest Statement

The authors declare that the research was conducted in the absence of any commercial or financial relationships that could be construed as a potential conflict of interest.

## References

[B1] Aiastui A. Pucciarelli M. G. García-Del Portillo F. (2010). Salmonella enterica serovar typhimurium invades fibroblasts by multiple routes differing from the entry into epithelial cells. *Infect. Immun.* 78 2700–2713 10.1128/IAI.01389-09 20368348 PMC2876547

[B2] Baisón-Olmo F. Cardenal-Muñoz E. Ramos-Morales F. (2012). PipB2 is a substrate of the *Salmonella* pathogenicity island 1-encoded type III secretion system. *Biochem. Biophys. Res. Commun.* 423 240–246 10.1016/j.bbrc.2012.05.095 22640733

[B3] Bajaj V. Hwang C. Lee C. A. (1995). hilA is a novel ompR/toxR family member that activates the expression of *Salmonella typhimurium* invasion genes. *Mol. Microbiol.* 18 715–727 10.1111/j.1365-2958.1995.mmi_18040715.x 8817493

[B4] Bajaj V. Lucas R. L. Hwang C. Lee C. A. (1996). Co-ordinate regulation of *Salmonella typhimurium* invasion genes by environmental and regulatory factors is mediated by control of hilA expression. *Mol. Microbiol.* 22 703–714 10.1046/j.1365-2958.1996.d01-1718.x 8951817

[B5] Bakowski M. A. Braun V. Brumell J. H. (2008). Salmonella-containing vacuoles: directing traffic and nesting to grow. *Traffic* 9 2022–2031 10.1111/j.1600-0854.2008.00827.x 18778407

[B6] Bijlsma J. J. Groisman E. A. (2005). The PhoP/PhoQ system controls the intramacrophage type three secretion system of *Salmonella enterica*. *Mol. Microbiol.* 57 85–96 10.1111/j.1365-2958.2005.04668.x 15948951

[B7] Boyle E. C. Brown N. F. Finlay B. B. (2006). Salmonella enterica serovar Typhimurium effectors SopB, SopE, SopE2 and SipA disrupt tight junction structure and function. *Cell Microbiol.* 8 1946–1957 10.1111/j.1462-5822.2006.00762.x 16869830

[B8] Brown N. F. Coombes B. K. Bishop J. L. Wickham M. E. Lowden M. J. Gal-Mor O. (2011). Salmonella phage ST64B encodes a member of the SseK/NleB effector family. *PLoS ONE* 6:e17824 10.1371/journal.pone.0017824 PMC3060822 21445262

[B9] Brumell J. H. Kujat-Choy S. Brown N. F. Vallance B. A. Knodler L. A. Finlay B. B. (2003). SopD2 is a novel type III secreted effector of *Salmonella typhimurium* that targets late endocytic compartments upon delivery into host cells. *Traffic* 4 36–48 10.1034/j.1600-0854.2003.40106.x 12535274

[B10] Buckner M. M. Croxen M. A. Arena E. T. Finlay B. B. (2011). A comprehensive study of the contribution of *Salmonella enterica* serovar Typhimurium SPI2 effectors to bacterial colonization, survival, and replication in typhoid fever, macrophage, and epithelial cell infection models. *Virulence* 2 208–216 10.4161/viru.2.3.15894 21540636 PMC3149682

[B11] Bullock W. O. Fernandez J. M. Short J. M. (1987). XL1-BLUE: a high efficiency plasmid transforming RecA *Escherichia coli* strain with beta-galactosidase selection. *BioTechniques* 5 376–379.

[B12] Cardenal-Muñoz E. Ramos-Morales F. (2011). Analysis of the expression, secretion and translocation of the *Salmonella enterica* type III secretion system effector SteA. *PLoS ONE* 6:e26930 10.1371/journal.pone.0026930 PMC3203157 22046414

[B13] Cardenal-Muñoz E. Ramos-Morales F. (2013). DsbA and MgrB regulate steA expression through the two-component system PhoQ/PhoP in *Salmonella enterica*. *J. Bacteriol.* 195 2368–2378 10.1128/JB.00110-13 23504014 PMC3650522

[B14] Chan R. K. Botstein D. Watanabe T. Ogata Y. (1972). Specialized transduction of tetracycline resistance by phage P22 in *Salmonella typhimurium*. II. Properties of a high-frequency-transducing lysate. *Virology* 50 883–898 10.1016/0042-6822(72)90442-4 4565618

[B15] Chen Z. Jiang X. (2014). Microbiological safety of chicken litter or chicken litter-based organic fertilizers: a review. *Agriculture* 4 1–29 10.3390/agriculture4010001

[B16] Cherepanov P. P. Wackernagel W. (1995). Gene disruption in *Escherichia coli*: TcR and KmR cassettes with the option of Flp-catalyzed excision of the antibiotic-resistance determinant. *Gene* 158 9–14 10.1016/0378-1119(95)00193-A 7789817

[B17] Cirillo D. M. Valdivia R. H. Monack D. M. Falkow S. (1998). Macrophage-dependent induction of the *Salmonella* pathogenicity island 2 type III secretion system and its role in intracellular survival. *Mol. Microbiol.* 30 175–188 10.1046/j.1365-2958.1998.01048.x 9786194

[B18] Cordero-Alba M. Ramos-Morales F. (2014). Patterns of expression and translocation of the ubiquitin ligase SlrP in *Salmonella enterica* serovar Typhimurium. *J. Bacteriol.* 196 3912–3922 10.1128/JB.02158-14 25182488 PMC4248824

[B19] Datsenko K. A. Wanner B. L. (2000). One-step inactivation of chromosomal genes in *Escherichia coli* K-12 using PCR products. *Proc. Natl. Acad. Sci. U.S.A.* 97 6640–6645 10.1073/pnas.120163297 10829079 PMC18686

[B20] Ellermeier C. D. Janakiraman A. Slauch J. M. (2002). Construction of targeted single copy lac fusions using lambda Red and FLP-mediated site-specific recombination in bacteria. *Gene* 290 153–161 10.1016/S0378-1119(02)00551-6 12062810

[B21] Fink S. L. Cookson B. T. (2007). Pyroptosis and host cell death responses during *Salmonella* infection. *Cell Microbiol* 9 2562–2570 10.1111/j.1462-5822.2007.01036.x 17714514

[B22] Galán J. E. Curtiss R. III. (1989). Cloning and molecular characterization of genes whose products allow *Salmonella typhimurium* to penetrate tissue culture cells. *Proc. Natl. Acad. Sci. U.S.A.* 86 6383–6387 10.1073/pnas.86.16.6383 2548211 PMC297844

[B23] Gal-Mor O. Elhadad D. Deng W. Rahav G. Finlay B. B. (2011). The *Salmonella enterica* PhoP directly activates the horizontally acquired SPI-2 gene sseL and is functionally different from a *S. bongori* ortholog. *PLoS ONE* 6:e20024 10.1371/journal.pone.0020024 PMC3098285 21625519

[B24] Gantois I. Ducatelle R. Pasmans F. Haesebrouck F. Hautefort I. Thompson A. (2006). Butyrate specifically down-regulates *Salmonella* pathogenicity island 1 gene expression. *Appl. Environ. Microbiol.* 72 946–949 10.1128/AEM.72.1.946-949.2006 16391141 PMC1352287

[B25] García-Calderón C. B. Casadesús J. Ramos-Morales F. (2007). Rcs and PhoPQ regulatory overlap in the control of *Salmonella enterica* virulence. *J. Bacteriol.* 189 6635–6644 10.1128/JB.00640-07 17616593 PMC2045174

[B26] García-Calderón C. B. García-Quintanilla M. Casadesús J. Ramos-Morales F. (2005). Virulence attenuation in *Salmonella enterica rcsC* mutants with constitutive activation of the Rcs system. *Microbiology* 151 579–588 10.1099/mic.0.27520-0 15699206

[B27] García Véscovi E. Soncini F. C. Groisman E. A. (1996). Mg2+ as an extracellular signal: environmental regulation of *Salmonella* virulence. *Cell* 84 165–174 10.1016/S0092-8674(00)81003-X 8548821

[B28] Gong H. Su J. Bai Y. Miao L. Kim K. Yang Y. (2009). Characterization of the expression of *Salmonella* Type III secretion system factor PrgI, SipA, SipB, SopE2, SpaO, and SptP in cultures and in mice. *BMC Microbiol.* 9:73 10.1186/1471-2180-9-73 PMC2678129 19371445

[B29] Groisman E. A. Chiao E. Lipps C. J. Heffron F. (1989). Salmonella typhimurium phoP virulence gene is a transcriptional regulator. *Proc. Natl. Acad. Sci. U.S.A.* 86 7077–7081 10.1073/pnas.86.18.7077 2674945 PMC297997

[B30] Guiney D. G. Lesnick M. (2005). Targeting of the actin cytoskeleton during infection by *Salmonella* strains. *Clin. Immunol.* 114 248–255 10.1016/j.clim.2004.07.014 15721835

[B31] Gyles C. Boerlin P. (2014). Horizontally transferred genetic elements and their role in pathogenesis of bacterial disease. *Vet. Pathol.* 51 328–340 10.1177/0300985813511131 24318976

[B32] Habyarimana F. Sabag-Daigle A. Ahmer B. M. (2014). The SdiA-regulated gene srgE encodes a type III secreted effector. *J. Bacteriol.* 196 2301–2312 10.1128/JB.01602-14 24727228 PMC4054179

[B33] Hanahan D. (1983). Studies on transformation of *Escherichia coli* with plasmids. *J. Mol. Biol.* 166 557–580 10.1016/S0022-2836(83)80284-8 6345791

[B34] Haneda T. Ishii Y. Shimizu H. Ohshima K. Iida N. Danbara H. (2012). Salmonella type III effector SpvC, a phosphothreonine lyase, contributes to reduction in inflammatory response during intestinal phase of infection. *Cell. Microbiol.* 14 485–499 10.1111/j.1462-5822.2011.01733.x 22188134

[B35] Hautefort I. Thompson A. Eriksson-Ygberg S. Parker M. L. Lucchini S. Danino V. (2008). During infection of epithelial cells *Salmonella enterica* serovar *typhimurium* undergoes a time-dependent transcriptional adaptation that results in simultaneous expression of three type 3 secretion systems. *Cell. Microbiol.* 10 958–984 10.1111/j.1462-5822.2007.01099.x 18031307 PMC2343689

[B36] Hensel M. Shea J. E. Waterman S. R. Mundy R. Nikolaus T. Banks G. (1998). Genes encoding putative effector proteins of the type III secretion system of *Salmonella* pathogenicity island 2 are required for bacterial virulence and proliferation in macrophages. *Mol. Microbiol.* 30 163–174 10.1046/j.1365-2958.1998.01047.x 9786193

[B37] Jones M. A. Wood M. W. Mullan P. B. Watson P. R. Wallis T. S. Galyov E. E. (1998). Secreted effector proteins of *Salmonella* dublin act in concert to induce enteritis. *Infect. Immun.* 66 5799–57804. 9826357 10.1128/iai.66.12.5799-5804.1998 PMC108733

[B38] Knodler L. A. (2015). Salmonella enterica: living a double life in epithelial cells. *Curr. Opin. Microbiol.* 23C 23–31 10.1016/j.mib.2014.10.010 25461569

[B39] Knodler L. A. Vallance B. A. Celli J. Winfree S. Hansen B. Montero M. (2010). Dissemination of invasive *Salmonella* via bacterial-induced extrusion of mucosal epithelia. *Proc. Natl. Acad. Sci. U.S.A.* 107 17733–17738 10.1073/pnas.1006098107 20876119 PMC2955089

[B40] Kroger C. Dillon S. C. Cameron A. D. Papenfort K. Sivasankaran S. K. Hokamp K. (2012). The transcriptional landscape and small RNAs of *Salmonella enterica* serovar Typhimurium. *Proc. Natl. Acad. Sci. U.S.A.* 109 E1277–E1286 10.1073/pnas.1201061109 22538806 PMC3356629

[B41] Kujat Choy S. L. Boyle E. C. Gal-Mor O. Goode D. L. Valdez Y. Vallance B. A. (2004). SseK1 and SseK2 are novel translocated proteins of *Salmonella enterica* serovar typhimurium. *Infect. Immun.* 72 5115–5125 10.1128/IAI.72.9.5115-5125.2004 15322005 PMC517430

[B42] Lawhon S. D. Maurer R. Suyemoto M. Altier C. (2002). Intestinal short-chain fatty acids alter *Salmonella* typhimurium invasion gene expression and virulence through BarA/SirA. *Mol. Microbiol.* 46 1451–1464 10.1046/j.1365-2958.2002.03268.x 12453229

[B43] Liao A. P. Petrof E. O. Kuppireddi S. Zhao Y. Xia Y. Claud E. C. (2008). Salmonella type III effector AvrA stabilizes cell tight junctions to inhibit inflammation in intestinal epithelial cells. *PLoS ONE* 3:e2369 10.1371/journal.pone.0002369 PMC2408728 18523661

[B44] Lin D. Rao C. V. Slauch J. M. (2008). The *Salmonella* SPI1 type three secretion system responds to periplasmic disulfide bond status via the flagellar apparatus and the RcsCDB system. *J. Bacteriol.* 190 87–97 10.1128/JB.01323-07 17951383 PMC2223759

[B45] López-Garrido J. Casadesús J. (2010). Regulation of *Salmonella enterica* pathogenicity island 1 by DNA adenine methylation. *Genetics* 184 637–649 10.1534/genetics.109.108985 20008574 PMC2845334

[B46] Macián F. Pérez-Roger I. Armengod M. E. (1994). An improved vector system for constructing transcriptional lacZ fusions: analysis of regulation of the dnaA, dnaN, recF and gyrB genes of *Escherichia coli*. *Gene* 145 17–24 10.1016/0378-1119(94)90317-4 8045420

[B47] Maloy S. R. (1990). *Experimental Techniques in Bacterial Genetics*. Boston, MA: Jones & Barlett.

[B48] Mazurkiewicz P. Thomas J. Thompson J. A. Liu M. Arbibe L. Sansonetti P. (2008). SpvC is a *Salmonella* effector with phosphothreonine lyase activity on host mitogen-activated protein kinases. *Mol. Microbiol.* 67 1371–1383 10.1111/j.1365-2958.2008.06134.x 18284579 PMC2268955

[B49] Miao E. A. Freeman J. A. Miller S. I. (2002). Transcription of the SsrAB regulon is repressed by alkaline pH and is independent of PhoPQ and magnesium concentration. *J. Bacteriol.* 184 1493–1497 10.1128/JB.184.5.1493-1497.2002 11844786 PMC134869

[B50] Miao E. A. Miller S. I. (2000). A conserved amino acid sequence directing intracellular type III secretion by *Salmonella* typhimurium. *Proc. Natl. Acad. Sci. U.S.A.* 97 7539–7544 10.1073/pnas.97.13.7539 10861017 PMC16581

[B51] Miao E. A. Scherer C. A. Tsolis R. M. Kingsley R. A. Adams L. G. Baumler A. J. (1999). Salmonella typhimurium leucine-rich repeat proteins are targeted to the SPI1 and SPI2 type III secretion systems. *Mol. Microbiol.* 34 850–864 10.1046/j.1365-2958.1999.01651.x 10564523

[B52] Miller J. H. (1972). *Experiments in Molecular Genetics*. Cold Spring Harbor, NY: Cold Spring Harbor Laboratory Press.

[B53] Miller S. I. Mekalanos J. J. (1990). Constitutive expression of the phoP regulon attenuates *Salmonella* virulence and survival within macrophages. *J. Bacteriol.* 172 2485–2490. 2185222 10.1128/jb.172.5.2485-2490.1990 PMC208887

[B54] Montagne M. Martel A. Le Moual H. (2001). Characterization of the catalytic activities of the PhoQ histidine protein kinase of *Salmonella enterica* serovar Typhimurium. *J. Bacteriol.* 183 1787–1791 10.1128/JB.183.5.1787-1791.2001 11160113 PMC95067

[B55] Mouslim C. Delgado M. Groisman E. A. (2004). Activation of the RcsC/YojN/RcsB phosphorelay system attenuates *Salmonella* virulence. *Mol. Microbiol.* 54 386–395 10.1111/j.1365-2958.2004.04293.x 15469511

[B56] Mulder D. T. Mcphee J. B. Reid-Yu S. A. Stogios P. J. Savchenko A. Coombes B. K. (2015). Multiple histidines in the periplasmic domain of the *Salmonella enterica* sensor kinase SsrA enhance signaling in response to extracellular acidification. *Mol. Microbiol.* 95 678–691 10.1111/mmi.12895 25442048

[B57] Niemann G. S. Brown R. N. Gustin J. K. Stufkens A. Shaikh-Kidwai A. S. Li J. (2011). Discovery of novel secreted virulence factors from *Salmonella enterica* serovar Typhimurium by proteomic analysis of culture supernatants. *Infect. Immun.* 79 33–43 10.1128/IAI.00771-10 20974834 PMC3019877

[B58] Ochman H. Soncini F. C. Solomon F. Groisman E. A. (1996). Identification of a pathogenicity island required for *Salmonella* survival in host cells. *Proc. Natl. Acad. Sci. U.S.A.* 93 7800–7804 10.1073/pnas.93.15.7800 8755556 PMC38828

[B59] Prieto A. I. Ramos-Morales F. Casadesús J. (2004). Bile-induced DNA damage in *Salmonella enterica*. *Genetics* 168 1787–1794 10.1534/genetics.104.031062 15611156 PMC1448704

[B60] Ramos-Morales F. (2012). Impact of *Salmonella enterica* type III secretion system effectors on the eukaryotic host cell. *ISRN Cell Biol.* 2012:787934 10.5402/2012/787934

[B61] Ramos-Morales F. Cardenal-Muñoz E. Cordero-Alba M. Baisón-Olmo F. (2015). Generation and use of site-directed chromosomal cyaA’ translational fusions in *Salmonella enterica*. *Methods Mol. Biol.* 1225 93–104 10.1007/978-1-4939-1625-2_6 25253250

[B62] Schechter L. M. Lee C. A. (2001). AraC/XylS family members, HilC and HilD, directly bind and derepress the *Salmonella* typhimurium hilA promoter. *Mol. Microbiol.* 40 1289–1299 10.1046/j.1365-2958.2001.02462.x 11442828

[B63] Schmieger H. (1972). Phage P22-mutants with increased or decreased transduction abilities. *Mol. Gen. Genet.* 119 75–88 10.1007/BF00270447 4564719

[B64] Schroeder N. Mota L. J. Meresse S. (2011). Salmonella-induced tubular networks. *Trends Microbiol.* 19 268–277 10.1016/j.tim.2011.01.006 21353564

[B65] Segura I. Casadesús J. Ramos-Morales F. (2004). Use of mixed infections to study cell invasion and intracellular proliferation of *Salmonella enterica* in eukaryotic cell cultures. *J. Microbiol. Methods* 56 83–91 10.1016/j.mimet.2003.09.004 14706753

[B66] Shea J. E. Hensel M. Gleeson C. Holden D. W. (1996). Identification of a virulence locus encoding a second type III secretion system in *Salmonella* typhimurium. *Proc. Natl. Acad. Sci. U.S.A.* 93 2593–2597 10.1073/pnas.93.6.2593 8637919 PMC39842

[B67] Steele-Mortimer O. (2008). The *Salmonella*-containing vacuole: moving with the times. *Curr. Opin. Microbiol.* 11 38–45 10.1016/j.mib.2008.01.002 18304858 PMC2577838

[B68] Tang Y. T. Gao R. Havranek J. J. Groisman E. A. Stock A. M. Marshall G. R. (2012). Inhibition of bacterial virulence: drug-like molecules targeting the *Salmonella enterica* PhoP response regulator. *Chem. Biol. Drug Des.* 79 1007–1017 10.1111/j.1747-0285.2012.01362.x 22339993 PMC3445336

[B69] Tsolis R. M. Kingsley R. A. Townsend S. M. Ficht T. A. Adams L. G. Baumler A. J. (1999). Of mice, calves, and men. Comparison of the mouse typhoid model with other *Salmonella* infections. *Adv. Exp. Med. Biol.* 473 261–274 10.1007/978-1-4615-4143-1_28 10659367

[B70] Uzzau S. Brown D. J. Wallis T. Rubino S. Leori G. Bernard S. (2000). Host adapted serotypes of *Salmonella enterica*. *Epidemiol. Infect.* 125 229–255 10.1017/S0950268899004379 11117946 PMC2869595

[B71] Uzzau S. Figueroa-Bossi N. Rubino S. Bossi L. (2001). Epitope tagging of chromosomal genes in *Salmonella*. *Proc. Natl. Acad. Sci. U.S.A.* 98 15264–15269 10.1073/pnas.261348198 11742086 PMC65018

[B72] Winson M. K. Swift S. Hill P. J. Sims C. M. Griesmayr G. Bycroft B. W. (1998). Engineering the luxCDABE genes from *Photorhabdus luminescens* to provide a bioluminescent reporter for constitutive and promoter probe plasmids and mini-Tn5 constructs. *FEMS Microbiol. Lett.* 163 193–202 10.1111/j.1574-6968.1998.tb13045.x 9673022

[B73] Worley M. J. Ching K. H. Heffron F. (2000). Salmonella SsrB activates a global regulon of horizontally acquired genes. *Mol. Microbiol.* 36 749–761 10.1046/j.1365-2958.2000.01902.x 10844662

[B74] Zhang Y. Higashide W. M. Mccormick B. A. Chen J. Zhou D. (2006). The inflammation-associated *Salmonella* SopA is a HECT-like E3 ubiquitin ligase. *Mol. Microbiol.* 62 786–793 10.1111/j.1365-2958.2006.05407.x 17076670

